# 0034. Changes of TLR2, TLR4, MYD88 MRNA expressions on peripheral blood mononuclear cell in severe sepsis patients during treatment with thymosin α1

**DOI:** 10.1186/2197-425X-2-S1-O7

**Published:** 2014-09-26

**Authors:** Z Tang, J Wu, J Chen, B Ouyang, M Chen, X Guan

**Affiliations:** The First Affiliated Hospital of Sun Yat-sen University, Department of Surgical Intensive Care Unit, Guangzhou, China

## Introduction

Severe sepsis is associated with a high mortality rate despite implementation of guidelinerecommendations. Thymosin alpha 1 (Ta1) has been used to treat severe sepsis as a promising beneficial immunomodulatory drug, but its mechanism remains unclear.

## Objectives

To examine the TLR2, TLR4 and MyD88 mRNA expressions on human peripheral blood mononuclear cells during treatment.

## Methods

A prospective randomized control trial was designed. Fifty-four patients with severe sepsis were enrolled and randomized into thymosin group (26 cases, treated with thymosin alpha 1) and control group (28 cases). TLR2, TLR4 and MyD88 mRNA were tested by RT-PCR on the day of enrollment, day 3 and day 7 after treatment in both groups, 28 day mortality in these patients was analyzed.

## Results

There was no statistical significance between 28 day mortality rate in the control group and that of thymosin group(35.7% vs 23.1%, P=0.310). TLR2, TLR4 and MyD88 mRNA expressions in the thymosin group were respectively increased on enrollment day, day 3 and day 7, however, this increasing trend was not found in the control group. On day 3, TLR2 and TLR4 mRNA expressions in the thymosin group were higher than those in the control group (2.31±0.79 vs 1.83±0.51, 7.31±0.79 vs 6.55±0.92, P<0.05). On day 7, TLR2, TLR4 and MyD88 mRNA expressions in the thymosin group were higher than those in the control group (2.75±1.17 vs 1.63±0.36; 7.75±1.03 vs 6.39±0.72; 4.26±0.77 vs 3.77±0.68, P<0.05).

## Conclusions

Thymosin alpha 1 may increase TLR2,TLR4 and MyD88 mRNA expressions in severe sepsis patients.
